# Chemical Coaxing of Mesenchymal Stromal Cells by Drug Repositioning for Nestin Induction

**DOI:** 10.3390/ijms25158006

**Published:** 2024-07-23

**Authors:** Sun-Ung Lim, Dae-Won Lee, Jung-Ho Kim, Young-Ju Kang, In-Yong Kim, Il-Hoan Oh

**Affiliations:** 1 Catholic High-Performance Cell Therapy Center & Department of Medical Life Science, College of Medicine, The Catholic University of Korea, 222, Banpo-Daero, Seocho-Gu, Seoul 06591, Republic of Korea; woong@catholic.ac.kr (S.-U.L.); lovebauoo@catholic.ac.kr (D.-W.L.); iykim@catholic.ac.kr (I.-Y.K.); 2 Regen Innopharm Inc., Seoul 06591, Republic of Korea; leeluleel@catholic.ac.kr (J.-H.K.); yjkang@regeninnopharm.com (Y.-J.K.)

**Keywords:** MSCs, chemical engineering, nestin, niche activity

## Abstract

Mesenchymal stromal cells (MSCs) display heterogeneity in origin and functional role in tissue homeostasis. Subsets of MSCs derived from the neural crest express nestin and serve as niches in bone marrow, but the possibility of coaxing MSCs into nestin-expresing cells for enhanced supportive activity is unclear. In this study, as an approach to the chemical coaxing of MSC functions, we screened libraries of clinically approved chemicals to identify compounds capable of inducing nestin expression in MSCs. Out of 2000 clinical compounds, we chose vorinostat as a candidate to coax the MSCs into neural crest-like fates. When treated with vorinostat, MSCs exhibited a significant increase in the expression of genes involved in the pluripotency and epithelial–mesenchymal transition (EMT), as well as nestin and CD146, the markers for pericytes. In addition, these nestin-induced MSCs exhibited enhanced differentiation towards neuronal cells with the upregulation of neurogenic markers, including SRY-box transcription factor 2 (Sox2), SRY-box transcription factor 10 (Sox10) and microtubule associated protein 2 (Map2) in addition to nestin. Moreover, the coaxed MSCs exhibited enhanced supporting activity for hematopoietic progenitors without supporting leukemia cells. These results demonstrate the feasibility of the drug repositioning of MSCs to induce neural crest-like properties through the chemical coaxing of cell fates.

## 1. Introduction

Mesenchymal stromal cells (MSCs) are a type of non-hematopoietic adherent cells derived from bone marrow (BM), adipose tissue, or placental tissue. These cells possess the ability to differentiate into multiple tissue types, including bone, cartilage and adipose tissue [1,2,3,4,5]. Accumulating studies indicate that MSCs primarily facilitate tissue regeneration through paracrine mechanisms, which exhibit both anti-apoptotic and anti-fibrotic properties [6] and promote the regeneration of endogenous stem cells such as hematopoietic stem cells (HSCs), neuronal stem cells and other tissue-specific stem cells [7,8]. Consequently, MSCs have been extensively investigated in clinical cell therapy trials, with over 425 trials for 2016–2023, conducted using ex vivo expanded MSCs for tissue regeneration (from a search for mesenchymal stem cells at www.clinicaltrials.gov (accessed on 8 July 2024)). 

However, MSCs exhibit significant heterogeneity, and only certain primitive subsets have the capacity to form colonies (colony-forming unit-fibroblasts, CFU-Fs) and support endogenous stem cells. In BM, only these primitive MSC subsets with colony-forming potential contribute to the stem cell niches [9,10]. Subsequent studies have identified MSC subsets expressing markers such as nestin [11], leptin receptor [12] or prx-1 [13], which are enriched with CFU-F and serve as key HSC-supporting niche cells. Furthermore, in ex vivo cultured MSCs, there is a positive correlation between progenitors and their ability to support HSC self-renewal, despite functional differences between cultured and freshly isolated MSCs [14]. 

Recently, developmental origin was shown to comprise another distinction for functional characteristics in MSCs. Neural crest-derived stem cells, known for their extensive plasticity as multipotent stem cells/progenitors, are capable of differentiating into multiple lineages, including MSCs in the cranial regions and MSCs in BM [15,16,17]. This makes neural crest-derived stem cells particularly relevant for studies related to neural differentiation and regeneration. Moreover, recent studies showed that MSCs from the mesodermal sclerotome in the BM primarily contribute to the formation of bone and adipose tissues, whereas neural crest-derived MSCs expressing nestin are involved in the development of Schwann’s cells and BM niche cells that support HSC self-renewal. This reveals the differences in MSC functions with ontological heterogeneity, distinguishing them from mesoderm-derived MSCs [18,19]. 

Among these potential phenotypic discrepancies, MSCs expressing nestin have been associated with specific properties as primitive subsets, serving as niche cells supporting the stem cells [11]. Nestin is primarily known as a marker for neural stem/progenitor cells (NSCs/NPCs) in the nervous system, where it plays a crucial role in maintaining neural progenitor cells during development and adulthood [11,20]. However, nestin expression is not limited to neural cells. In particular, nestin has been identified as a marker for both peri-arteriolar and peri-sinusoidal pericytes in the BM [21]. Accordingly, there is interest in the characteristics of these nestin-expressing MSCs as a phenotype of pericyte that can serve as niche cells stimulating the self-renewal and/or proliferation of hematopoietic stem cells. Despite this interest, the functional changes in MSCs that could be caused by the induction of nestin remains unclear. 

In this study, we sought to identify the cellular characteristics of MSCs with nestin expression induced by chemical engineering. To achieve this, we screened a library of 2000 clinically approved chemicals to identify a compound capable of inducing nestin expression in MSCs. From this screening, we identified vorinostat, a chemical that induces nestin expression and drives the MSCs towards neural crest-like stem cells exhibiting higher neuronal differentiation and enhanced support on hematopoietic progenitors. Our findings suggest that drug repositioning could serve as a strategy for modulating MSCs to enhance specific desired cellular functions.

## 2. Results

### 2.1. Chemical Repositioning of Clinically Approved Drugs for Nestin Induction

**Figure 1:**
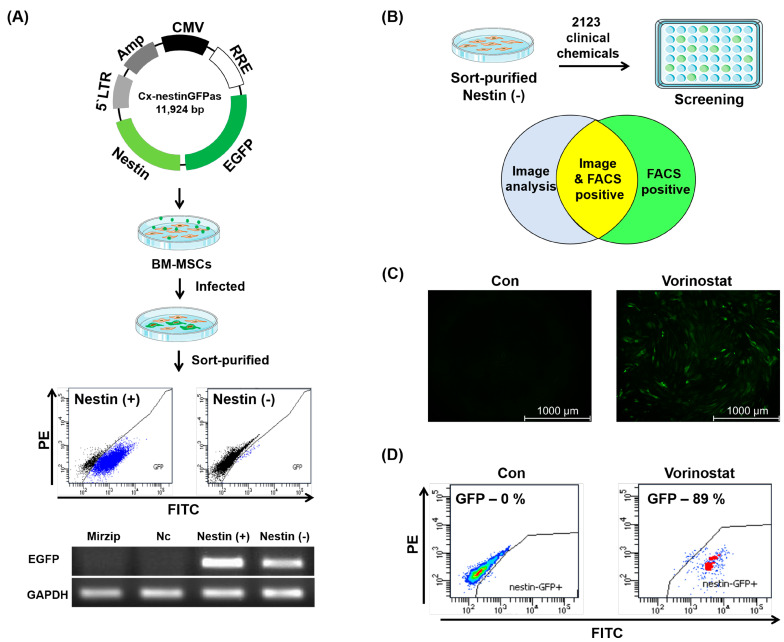
Chemical repositioning of clinically approved drugs for nestin induction. (**A**) Schematic illustrating a reporter system for nestin expression. This vector contains the nestin promoter to drive the expression of green fluorescent protein (GFP). The infected BM-MSCs at the basal level of GFP expression were sort-purified as nestin (−), confirmed by genomic PCR. Blue color indicates the GFP (+) MSCs. (**B**) Screening chemicals that induce nestin expression. For nestin induction, 2123 clinically approved chemicals (10 μM) were screened in the nestin-reporting MSCs through analysis using Image J and flow cytometry. (**C**,**D**) Vorinostat-induced nestin expression in the MSCs as confirmed by fluorescence microscopy and FACS. Pseudo-color represents cellular density.

Given that nestin expression in MSCs is associated with their enhanced therapeutic potential and specific subsets of MSCs [18,20,22], we were interested in exploring the potential of chemical engineering to upregulate the expression of nestin in MSCs. To establish a system for screening chemicals that induce nestin expression, we first created a lentiviral vector designed to serve as a reporter for nestin expression. This vector utilizes the nestin promoter to drive the expression of green fluorescent protein (GFP), as shown in Figure 1A. Primary BM-derived MSCs were infected with this reporter vector. Upon infection with the reporter vector, the endogenous basal level expression of nestin was observed in human BM-derived MSCs without any treatment. To overcome this limitation, we sort-purified MSC populations that were negative for basal levels of GFP (Nestin (−)), and the presence of the reporter vector in these sorted cells was confirmed by genomic PCR specific for the transgene of the vector (Figure 1A). The purified and transduced population of human MSCs (referred to as nestin-reporting MSCs) was employed for screening the chemical library (Figure 1B). We obtained 2123 chemicals, approved for clinical use by the U.S. Food and Drug Administration (FDA), from Korea Chemical Bank of the Korea Research Institute of Chemical Technology and screened them for their ability to induce nestin expression in MSCs. Through analysis using Image J 1.52r and flow cytometry, we identified five chemicals capable of converting GFP (−) MSCs to GFP (+) MSCs (Table S1). Among those compounds, we focused on vorinostat (also known as suberoylanilide-hydroxamic acid: SAHA), the first FDA-approved histone deacetylase (HDAC) inhibitor and a member of a larger class of compounds that inhibit HDAC, considering previous studies demonstrating the influence of HDACs on the cellular behavior of several stem cell populations [23,24,25,26]. Due to moderate cellular toxicity at higher concentrations of chemicals (>10 μM) during the initial chemical screening, subsequent studies on the MSCs were performed at lower concentrations (0.5, 1.0 and 5.0 μM) to optimize nestin expression in MSCs and ensure their survival (5 μM) (Figure S1). 

The treatment of vorinostat in GFP (−) MSCs confirmed vorinostat-induced nestin expression in the MSCs, comparable to non-treated MSCs (Figure 1C,D). 

### 2.2. Cellular Changes in Chemically Engineered Nestin-Expressing MSCs 

**Figure 2:**
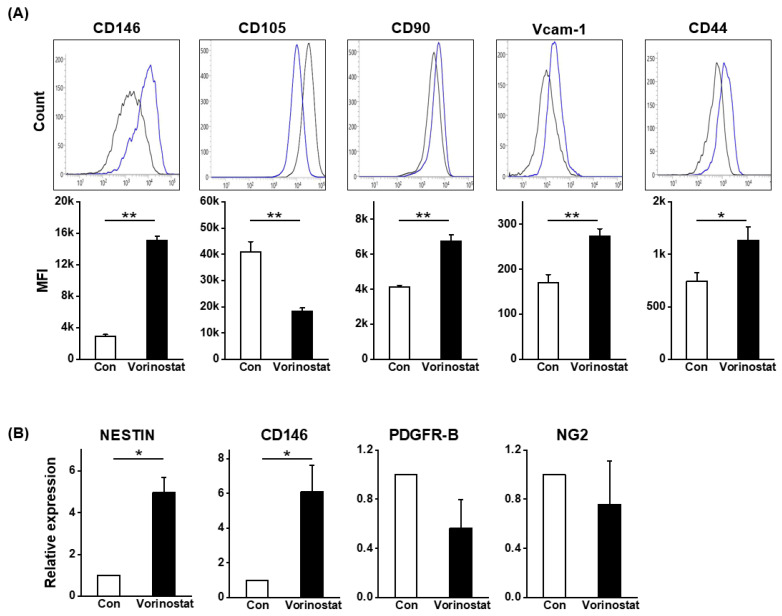
Vorinostat-induced changes in MSCs into pericyte-like phenotypes. (**A**) Flow cytometry analysis of MSC phenotypes by vorinostat treatment. Shown are the representative flowcytometry profile (upper) and mean fluorescence intensity (MFI) (lower) of each indicated molecule in vorinostat-treated (5 μM) MSCs, as blue-colored. (**B**) PCR analysis of vorinostat-induced changes in nestin- or pericyte-specific genes (CD146, PDGFR−B and NG2). Data are represented as mean ± SD. * *p* < 0.05, ** *p* < 0.01.

**Figure 3:**
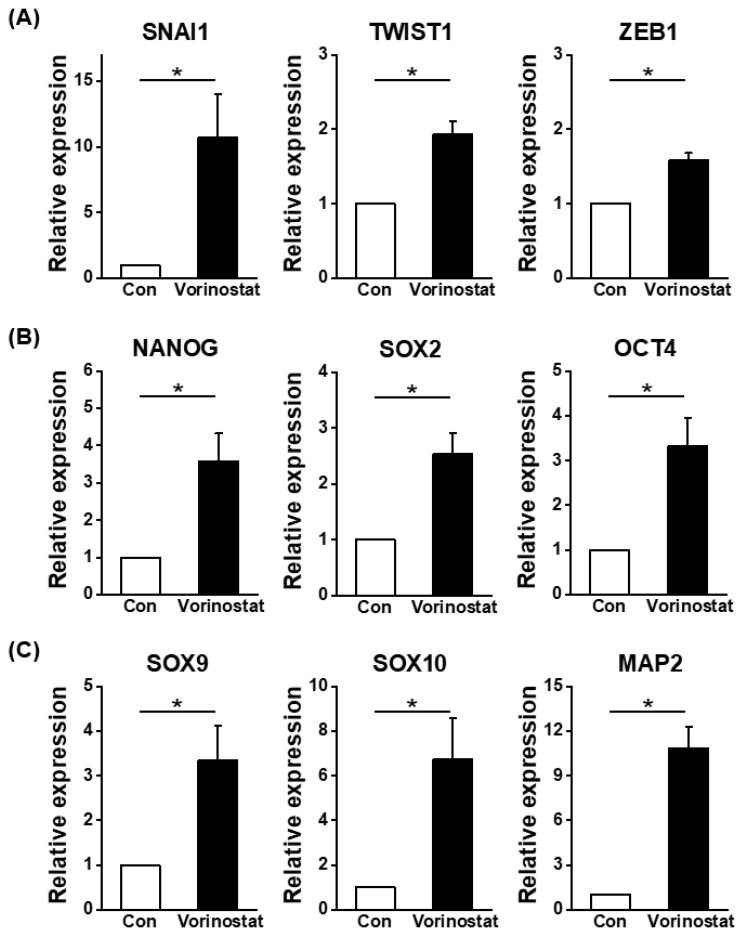
Expression changes in genes related to EMT, pluripotency and neuronal lineage in MSCs treated with vorinostat. (**A**) Quantitative PCR analysis of genes for EMT master regulatory genes (SNAIL-1, TWIST1 and ZEB-1) in vorinostat (5 μM)-treated MSCs. (**B**) Quantitative analysis for expression of stemness-related genes (NANOG, SOX2 and OCT4) in vorinostat-treated MSCs. (**C**) Quantitative analysis of expression of genes associated with neuronal lineages (SOX9, SOX10 and MAP2) in the basal culture in the absence of differentiation media. Data are represented as mean ± SD. * *p* < 0.05.

**Figure 4:**
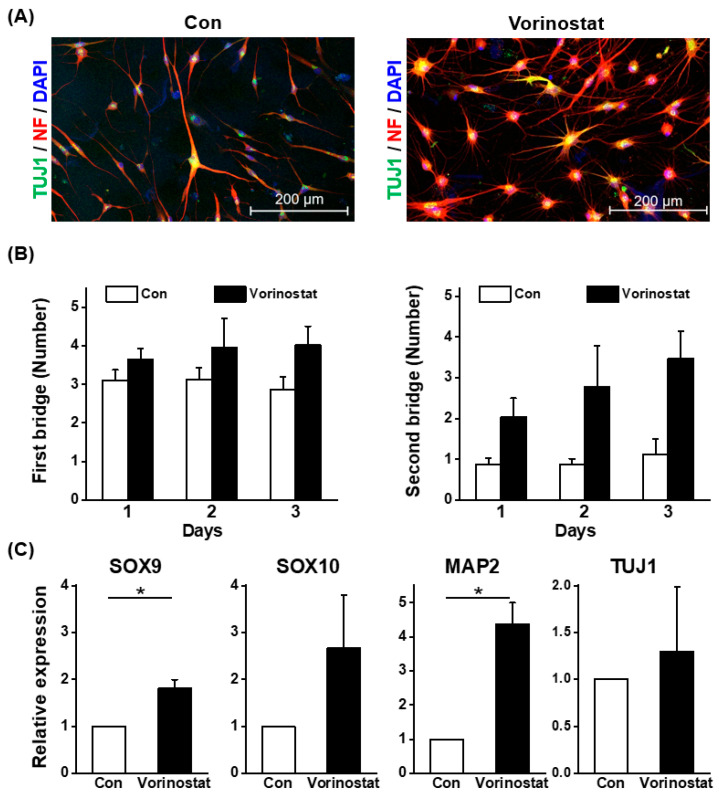
Effect of vorinostat on the differentiation of MSCs towards neuron-like cells. (**A**) Confocal images showing the expression of neurofilament and TUJ1 in MSCs at day 3 post-neuronal induction. (**B**) Quantification of the primary and secondary bridge numbers on dendritic outgrowth during neuronal differentiation. (**C**) Quantitative PCR analysis of neuronal lineage-specific genes (SOX9, SOX10, MAP2 and TUJ1) at day 3 post-neuronal induction. Data are represented as mean ± SD. * *p* < 0.05.

Human BM-derived MSCs were treated with vorinostat to assess its effects on their cellular characteristics during culture. The changes in the canonical phenotype of MSCs induced by vorinostat were examined. As shown in Figure 2A,B, vorinostat caused a significant induction of nestin and CD146, the pericyte markers for human MSCs [27,28,29], along with nestin expression, suggesting a pericyte-like change in MSCs. Meanwhile, other pericyte markers such as platelet derived growth factor receptor beta (PDGFR-B) and neuron-glial antigen 2 NG2 [30] did not differ between the control and treated MSCs (Figure 2B). Additionally, modest changes were observed in the expression levels of CD90, vascular cell adhesion molecule-1 (VCAM-1) and CD44 (Figure 2A), which are involved in cell–cell interaction [31] and colony formation [20]. These data show that vorinostat induces a phenotypical change in MSCs, leading them to adopt a pericyte-like phenotype. We have previously shown that pericyte-like changes in MSCs were associated with increased expression of stemness-related genes and an increased gradient for epithelial–mesenchymal transition (EMT) [32]. Consequently, focusing on the changes towards a pericyte-like phenotype, we examined changes in the expression levels of molecules involved in EMT or stemness. As shown in Figure 3A, an increase in EMT master regulatory genes (snail family transcriptional repressor-1 (SNAI1), twist-related protein 1 (TWIST1), and zinc-finger E-box-binding homeobox 1 (ZEB1)) was observed. Similarly, stemness-related genes were induced, supported by the induction of NANOG, SOX2 and OCT4 (Figure 3B). Interestingly, the vorinostat-mediated induction of nestin caused the up-regulation of genes associated with neuronal lineages, such as SOX9, SOX10 and MAP2 (Figure 3C) [33]. These findings indicate that nestin induction in MSCs partially drives the cells towards a state that mimics the characteristics of neural crest-derived stem cells, where properties of neural stem cells are preserved with multipotent stem cell characteristics [34]. To further explore this possibility, we evaluated the potential of vorinostat-treated MSCs to differentiate into neuronal cells by exposing them to neuronal differentiation induction media for 3 days. As shown in Figure 4A, MSCs exhibited elevated expression levels of Beta III Tubulin (TUJ1) and neurofilament (NF) (Figure 4A). In particular, the number of dendritic outgrowths significantly increased, as evidenced by the greater numbers of primary and secondary bridges during neuronal differentiation (Figure 4B), suggesting that vorinostat-treated MSCs exhibit enhanced differentiation into mature neuronal cells. Additionally, Figure 3C show the increased expression of neuronal lineage-specific genes (SOX9, SOX10, MAP2 and TUJ1) at day 3 post-neuronal induction. Taken together, these results suggest that the chemical induction of nestin in MSCs by vorinostat drives them towards a neural crest-like state, characterized by a higher propensity for neuronal differentiation, the partial acquisition of stemness and characteristics of EMT.

### 2.3. Niche Cell Activity Induced by Vorinostat

**Figure 5:**
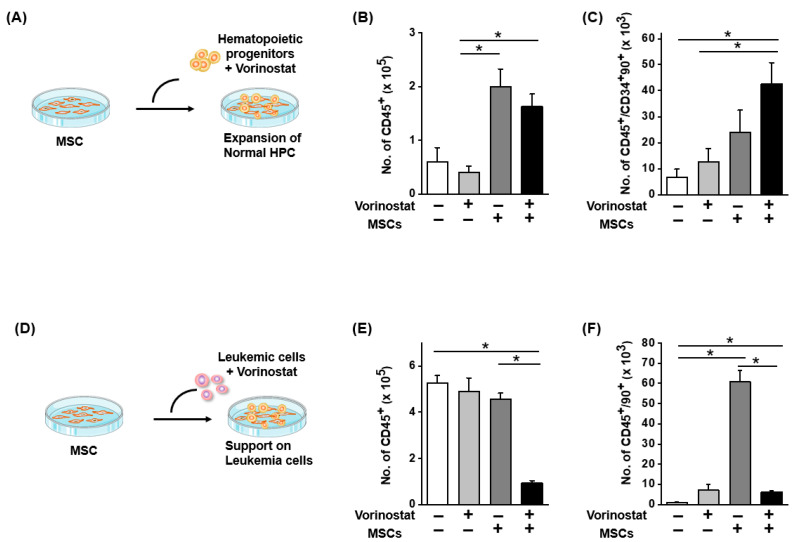
Distinct niche cell activity of MSCs on normal and leukemic cells induced by vorinostat. (**A**) Effects of vorinostat-treated MSCs on the support of normal hematopoietic progenitors. CD34+ cells from normal human umbilical cord blood were co-cultured with MSCs in the presence or absence of vorinostat. (**B**) Numbers of total hematopoietic cells (CD45+) in stroma-free co-culture or co-culture with MSCs in the presence or absence of vorinostat (0.5 μM) for 4 days. Mean cell numbers after culture are shown. (**C**) Number of primitive hematopoietic progenitors (CD45+CD34+90+) after co-culture with or without MSCs in the presence or absence of vorinostat (0.5 μM) for 4 days. Shown are numbers of CD34+90+CD45+ hematopoietic subsets after culture (n = 7, * *p* < 0.05). (**D**) Effects of vorinostat on the expansion of leukemia cells in the MSC co-culture. MOLM-14 leukemia cells were co-cultured with MSCs in the presence of vorinostat. (**E**) Total number of MOLM-14 leukemia cells (CD45+) after co-culture with or without MSCs in the presence or absence of vorinostat (0.5 μM) for 4 days. (**F**) Number of primitive leukemic cell subsets (CD45+90+) after co-culture with or without MSCs in the presence or absence of vorinostat (0.5 μM) for 4 days. (n = 3, * *p* < 0.05). Nestin (−) MSCs were used in this co-culture study.

Previous studies demonstrated that subsets of MSCs functioning as niches for stem cell support originate from the neural crest rather than the sclerotome [18]. Given our observation that the vorinostat-mediated induction of nestin expression caused neural crest-like changes in MSCs, we were interested in investigating whether these MSCs could exhibit enhanced niche activity to support stem cells. To examine this possibility, we first co-cultured human umbilical cord blood-derived CD34+ hematopoietic progenitor cells with MSCs in the presence or absence of vorinostat (Figure 5A). As shown in Figure 5B, the total numbers of hematopoietic cells (CD45+) after culture were not influenced by vorinostat in the presence or absence of mesenchymal stroma. Co-culture with MSCs led to an increase in total cell numbers, not influenced by the presence of vorinostat. In contrast, when assessing more primitive hematopoietic progenitor (CD34+CD90+CD45+) cells, a significant increase in their numbers was observed in the presence of mesenchymal stroma and vorinostat, whereas such increase was not seen with vorinostat co-culture with MSC alone (Figure 5C). This increase in primitive hematopoietic subsets observed in the presence of MSCs and vorinostat indicates that vorinostat exerted a stimulatory effect through its effects on MSCs. 

**Figure 6:**
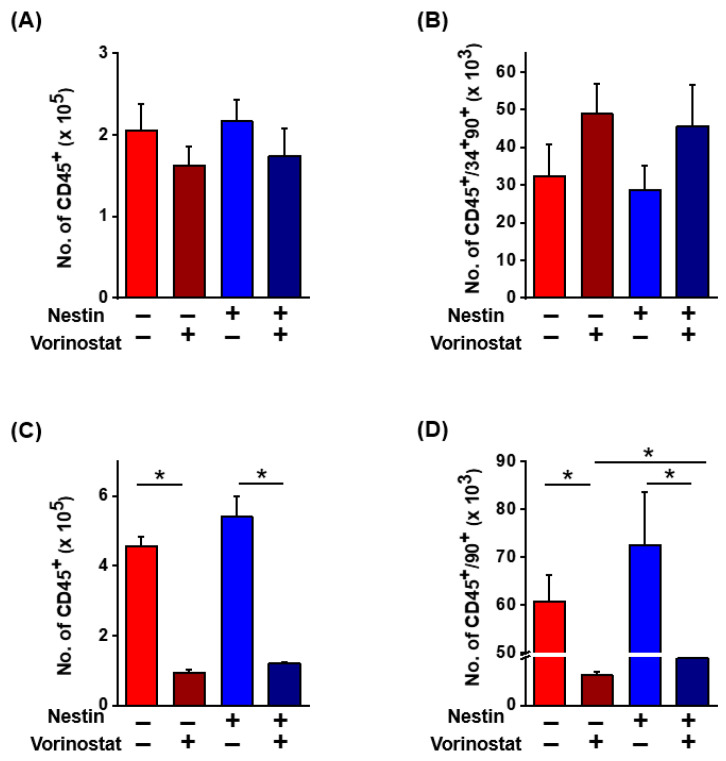
Comparisons of vorinostat’s effects on normal hematopoietic and leukemic cells in co-culture with nestin (−) and (+) MSCs. (**A**) Number of total hematopoietic cells (CD45+) after co-culture with nestin (−) or (+) MSCs in the presence or absence of vorinostat (0.5 μM) for 4 days. (**B**) Number of primitive hematopoietic progenitors (CD45+CD34+90+) after co-culture with nestin (−) or (+) MSCs in the presence or absence of vorinostat (0.5 μM) for 4 days. Shown are numbers of CD34+90+CD45+ subsets after co-culture (n = 7, * *p* < 0.05). Data on nestin (−) MSCs are derived from Figure 5 for comparison with cell numbers in nestin (+) cells. (**C**) Number of MOLM-14 leukemia cells (CD45+) after co-culture with nestin (−) or (+) MSCs in the presence or absence of vorinostat (0.5 μM) for 4 days. (**D**) Number of primitive leukemic cell subsets (CD45+90+) co-cultured with nestin (−) or (+) MSCs in the presence or absence of vorinostat (0.5 μM) for 4 days. (n = 3, * *p* < 0.05). Data on nestin (−) MSCs are derived from Figure 5 for comparison with data from nestin (+) cells.

Next, considering that vorinostat has been used clinically for the treatment of cutaneous T-cell lymphoma and myelodysplastic syndrome in combination with cytotoxic drugs [35,36,37], we were also interested in investigating whether vorinostat could exert opposing, inhibitory effects unlike its effects on normal HPCs. To explore this hypothesis, leukemia cells were co-cultured with MSCs in the presence or absence of vorinostat (Figure 5D). As shown in Figure 5E,F, a significant decrease in the numbers of total leukemia cell numbers (CD45+) and primitive leukemic cell subsets (CD45+90+) was observed. In contrast, the direct treatment of vorinostat on leukemic cells, without stromal co-culture, did not significantly affect cell numbers, implicating stroma-mediated inhibitory effects. Considering these opposite effects on normal and leukemic cell, we next questioned whether these effects are specific for nestin (−) subsets of MSCs and therefore examined the effects of vorinostat on nestin (+) MSCs that were sorted after lentiviral infection (Figure 1). As shown in Figure 6, both positive stimulatory effects on normal hematopoietic progenitors or inhibitory effects on leukemic or leukemic stem cell populations were similarly observed in co-culture with a nestin (+) population as in a nestin (−) MSC population. These results indicate that the chemical regulation of MSCs with vorinostat changes their niche activity in a manner that selectively promotes support on normal HSCs while inhibiting leukemia stem cells, similarly recapitulating the distinct microenvironmental effects on normal and leukemic cells [38,39]. 

## 3. Discussion

With the widespread utilization of MSCs for cell therapeutic application, clinical outcomes of MSC-based cell therapy are being accumulated, highlighting the necessity to enhance the therapeutic potency of MSCs and the specific control of their cellular behavior. Accordingly, various trials to modulate the functional aspects of MSCs have included preconditioning, genetic modifications or priming of the cells [40,41,42,43]. In addition, recent trials of chemical repositioning of approved drugs further extend the feasibility of coaxing the functional characteristics of MSC [44,45]. 

The suboptimal efficacy of MSC-based cell therapy could originate from the heterogeneity of the cell population, arising from variations in the source of MSCs, culture conditions or intrinsic biological properties [46,47,48]. Interestingly, functional heterogeneity is also observed among the MSCs of different developmental origin; i.e., MSCs originating from the neural crest contribute to Schwann’s cells and stem cell niche cells that can support the self-renewal of HSCs [18]. Consistent with this perspective, previous studies have shown that neural crest-derived stem cells (NCSCs) harbor extensive plasticity, serving as multipotent stem cells/progenitors [16] capable of differentiating into multiple lineages [17], including MSCs found in the cranial regions and other organs [15]. We therefore hypothesized that coaxing MSCs into an NCSC-like state could be a way to enhance their neuronal nature as well as their specific niche function in supporting stem cells. Nestin was chosen as a marker to reflect the functional changes in MSCs towards a more primitive cellular state, given that the subset of MSCs expressing nestin is associated with mesenchymal progenitors exhibiting colony-forming activity, as well as specific subsets of MSCs harboring niche activity [11,20,22]. 

Chemical repositioning, also known as drug repositioning, is a strategy that involves applying existing therapeutics to new disease indications. This approach has gained popularity due to the high cost and failure rate associated with developing new drugs [42]. Therefore, in this study, we aimed to find a chemical capable of inducing nestin expression among the 2130 clinically approved chemicals and identified vorinostat as one of such candidates, which was selected based on previous studies concerning the epigenetic regulation of stem cell states [25,26,49] and the effects of HDAC inhibitor on both normal and malignant stem cells [24]. While exploring the effects of vorinostat on MSCs, we observed no significant changes in the cell fates or canonical phenotypes of MSCs. However, we found a significant induction of CD146 along with nestin, both of which are markers for pericytes [11,20]. In addition, we observed an induction of EMT-related and stemness-related genes, implicating a certain level of functional changes toward a more primitive pericyte-like state. Another important difference in vorinostat-treated MSCs was an increased expression level of neurogenic factors and their enhanced ability to undergo differentiation into more mature neurons, exhibiting the significantly increased outgrowth of neurites. Taken together, these findings suggest that the induction of nestin in MSCs through vorinostat treatment drives the cells toward more primitive states, but closer to neuronal lineages, i.e., neural crest-like stem cells. 

Another intriguing aspect of nestin induction in MSCs relates to the observation that nestin-expressing cells represent a subset of MSCs functioning as niche cells [18,20], which prompted us to consider the possibility that the chemical repositioning of vorinostat on MSCs could influence their niche activity to support stem cells. Notably, vorinostat treatment caused MSCs to function as a stimulatory niche for normal HPCs while acting as an inhibitory niche for leukemia stem cells. The reasons for these opposite effects on normal and leukemia stem cells remain unclear. However, our recent findings suggest that microenvironmental stimuli for tissue regeneration are accompanied by the remodeling of mesenchymal niche cells, which undergo phenotypic changes and selectively support normal HSCs without aiding leukemia stem cells [25,38,39]. It is noteworthy that vorinostat is used for the treatment of myelodysplastic syndrome in combination with cytotoxic drugs [37]. Considering the contrasting effects of vorinostat-treated MSCs on normal and leukemic stem cells, its anti-leukemic properties while sparing normal counterparts could represent another mechanism for the drug’s therapeutic effects on myelodysplasia. 

In conclusion, our study suggests that vorinostat-induced chemical regulation may alter MSC function, potentially mimicking stimulatory regenerative environments by remodeling MSCs into neural crest-like cells. However, further investigation is needed to explore the molecular profiles and conduct a rigorous analysis of the stem cell population, including additional in vivo studies. Nevertheless, our findings raise the possibility that potential chemical repositioning could influence MSC-based cell therapeutic trials, either by enhancing MSCs’ differentiation potential or their paracrine supportive function for tissue regeneration. Further screening of suitable chemicals could open new avenues for such chemical repositioning of MSCs for cell therapeutic applications. 

## 4. Material and Methods

### 4.1. Cell Culture 

Cells in umbilical cord blood were obtained from the Seoul Metropolitan Government Public Cord Blood Bank. BM-MSCs were isolated from a healthy donor’s aspirates with informed consent and approval from the Institutional Review Board of the Catholic University of Korea and Seoul St. Mary’s Hospital, the Catholic University of Korea (MC19TNSI0012 and MC19SNSI0059). MSC cultures were established from the mononuclear cell fraction and maintained in low Dulbecco’s modified Eagle’s medium (Hyclone, Logan, UT, USA) containing 10% fetal bovine serum (Hyclone, Logan, UT, USA), L-glutamine (Gibco Life technologies, Grand Island, NY, USA) and antibiotics (Anti-Anti^TM^, Gibco Life technologies, NY, USA) in a humidified 5% CO_2_ environment at 37 °C. MOLM-14 was cultured in RPMI medium (Gibco Life technologies, NY, USA) with 10% FBS (Hyclone, Logan, UT, USA) according to the DSMZ information. 

### 4.2. Treatment of Chemicals 

A library of 2123 clinically approved chemicals was kindly provided by Korea Chemical Bank (http://www.chembank.org/ (accessed on 12 December 2019)) of the Korea Research Institute of Chemical Technology. Vorionstat (SAHA) was purchased from Sigma-Aldrich (SML0061, St. Louis, MO, USA) and dissolved in DMSO before use. 

### 4.3. RNA Extraction and Quantitative RT-PCR 

Total RNAs from MSCs were isolated with Trizol (Invitrogen, San Diego, CA, USA), and cDNAs were synthesized using superiorScript III mastermix (Enzynomics, Daejeon, Republic of Korea) according to the manufacturers’ instructions. Quantitative real-time PCR (qRT-PCR) was performed using SYBR Premix Ex Taq II (Takara, Japan). Data were normalized to HPRT as an internal control through the 2^−∆∆Ct^ method. The primer sequences used in this study are listed in Supplementary Table S2.

### 4.4. Co-Culture 

Human CD34+ cells were isolated from umbilical cord blood using a CD34 MicroBead kit (Miltenyl Biotec, Bergisch Gladbach, Germany). For co-culture experiments, MSCs were pretreated with mitomycin-C (10 ug/mL) for 1 h, washed, and co-cultured with purified CD34+ cells for 4 days in DMEM + 10% FBS in the presence of cytokine mixtures (20 ng/mL hSCF, 20 ng/mL hFlt3L, 4 ng/mL hIL-3, 4 ng/mL hIL-6, 4 ng/mL hG-CSF and 0.2 × 10^6^ M hydrocortisone (ProSpec-Tany TechnoGene Ltd., Rehovot, Israel)) in the presence or absence of 0.5 uM of vorinostat. For the phenotypic analysis of ex vivo expanded hematopoietic cells, co-cultured cells were stained with antibodies against CD45, CD34 and CD90 (BD Pharmingen, San Jose, CA, USA) and analyzed by flow cytometry, with gating applied to the hematopoietic (CD45+) population. 

### 4.5. Neural Differentiation 

For the neural differentiation of MSCs, cells were seeded in a fibronectin-precoated dish at 60–80% confluency. Neural differentiation was induced in neuronal induction media (PromoCell-C28015, Heidelberg, Germany) in the presence or absence of vorinostat. The induction was carried out for 3 or 7 days with medium changes every 48 h. 

### 4.6. Immunocytochemistry and Western Blotting 

MSCs were seeded onto fibronectin-coated chamber slides and fixed with cold methanol. For blocking, cells were incubated in 10% goat serum-containing PBS for 1h at room temperature. After thorough washing, chamber slides were incubated with primary antibodies against neurofilament (NF), TUJ1 (Abcam, Cambridge, MA, USA), followed by visualization using secondary antibodies. Fluorescent images were obtained using a confocal laser scanning microscope (LSM700; ZEISS International, Oberkochen, Germany) and analyzed using ZEISS ZEN 3.1 Imaging Software. Additionally, the expression levels of neuronal markers were assessed via Western blot analysis using antibodies against Sox2 and NESTIN (Abcam). Protein bands were visualized and quantified using a Luminescent Image Analysis System (LAS-4000; Fuji film, Tokyo, Japan), with quantification using Image J software. The antibodies used in this study are listed in Supplementary Table S3.

### 4.7. Flow Cytometry of Leukemic Cells and Mesenchymal Stromal Cells 

Murine leukemic cells were analyzed by flow cytometry using the following antibodies: MSC surface markers including CD145, CD105, CD90, Vcam-1 (CD106) and CD44 (BD Pharmingen, San Jose, CA, USA). The antibodies used in this study are listed in Supplementary Table S3.

### 4.8. Statistical Analysis 

Two-group comparisons were conducted using a two-tailed, unpaired *t*-test. All data are expressed as mean values ± SD in n replicates. Significance levels were indicated as * *p* < 0.05, ** *p* < 0.01 and *** *p* < 0.001. One-way ANOVA was used to analyze the differences among the means for three or more groups. When the ANOVA test showed significant differences between the groups, Tukey’s test was used as a post hoc test to find differences between specific groups. Significance levels were indicated as * *p* < 0.05. Statistical analysis was performed using GraphPad Prism software (version 8.0.1; GraphPad Software, San Diego, CA, USA).

## Data Availability

Data is contained within the article and supplementary material. The data presented in this study are available on request from the corresponding author.

## Supplementary Materials

The supporting information can be downloaded at: https://www.mdpi.com/article/10.3390/ijms25158006/s1.

## References

[1] Hoang D.M., Pham P.T., Bach T.Q., Ngo A.T.L., Nguyen Q.T., Phan T.T.K., Nguyen G.H., Le P.T.T., Hoang V.T., Forsyth N.R. (2022). Stem cell-based therapy for human diseases. Signal Transduct. Target. Ther..

[2] Pittenger M.F., Discher D.E., Peault B.M., Phinney D.G., Hare J.M., Caplan A.I. (2019). Mesenchymal stem cell perspective: Cell biology to clinical progress. NPJ Regen. Med..

[3] Keating A. (2006). Mesenchymal stromal cells. Curr. Opin. Hematol..

[4] Pittenger M.F., Mackay A.M., Beck S.C., Jaiswal R.K., Douglas R., Mosca J.D., Moorman M.A., Simonetti D.W., Craig S., Marshak D.R. (1999). Multilineage potential of adult human mesenchymal stem cells. Science.

[5] Prockop D.J. (1997). Marrow stromal cells as stem cells for nonhematopoietic tissues. Science.

[6] Caplan A.I., Correa D. (2011). The MSC: An injury drugstore. Cell Stem Cell.

[7] Murphy M.B., Moncivais K., Caplan A.I. (2013). Mesenchymal stem cells: Environmentally responsive therapeutics for regenerative medicine. Exp. Mol. Med..

[8] Frenette P.S., Pinho S., Lucas D., Scheiermann C. (2013). Mesenchymal stem cell: Keystone of the hematopoietic stem cell niche and a stepping-stone for regenerative medicine. Annu. Rev. Immunol..

[9] Chan C.K., Chen C.C., Luppen C.A., Kim J.B., DeBoer A.T., Wei K., Helms J.A., Kuo C.J., Kraft D.L., Weissman I.L. (2009). Endochondral ossification is required for haematopoietic stem-cell niche formation. Nature.

[10] Sacchetti B., Funari A., Michienzi S., Di Cesare S., Piersanti S., Saggio I., Tagliafico E., Ferrari S., Robey P.G., Riminucci M. (2007). Self-renewing osteoprogenitors in bone marrow sinusoids can organize a hematopoietic microenvironment. Cell.

[11] Mendez-Ferrer S., Michurina T.V., Ferraro F., Mazloom A.R., Macarthur B.D., Lira S.A., Scadden D.T., Ma’ayan A., Enikolopov G.N., Frenette P.S. (2010). Mesenchymal and haematopoietic stem cells form a unique bone marrow niche. Nature.

[12] Ding L., Saunders T.L., Enikolopov G., Morrison S.J. (2012). Endothelial and perivascular cells maintain haematopoietic stem cells. Nature.

[13] Greenbaum A., Hsu Y.M., Day R.B., Schuettpelz L.G., Christopher M.J., Borgerding J.N., Nagasawa T., Link D.C. (2013). CXCL12 in early mesenchymal progenitors is required for haematopoietic stem-cell maintenance. Nature.

[14] Mitchell J.B., McIntosh K., Zvonic S., Garrett S., Floyd Z.E., Kloster A., Di Halvorsen Y., Storms R.W., Goh B., Kilroy G. (2006). Immunophenotype of human adipose-derived cells: Temporal changes in stromal-associated and stem cell-associated markers. Stem Cells.

[15] Nagoshi N., Shibata S., Kubota Y., Nakamura M., Nagai Y., Satoh E., Morikawa S., Okada Y., Mabuchi Y., Katoh H. (2008). Ontogeny and multipotency of neural crest-derived stem cells in mouse bone marrow, dorsal root ganglia, and whisker pad. Cell Stem Cell.

[16] Trentin A., Glavieux-Pardanaud C., Le Douarin N.M., Dupin E. (2004). Self-renewal capacity is a widespread property of various types of neural crest precursor cells. Proc. Natl. Acad. Sci. USA.

[17] Stemple D.L., Anderson D.J. (1992). Isolation of a stem cell for neurons and glia from the mammalian neural crest. Cell.

[18] Isern J., Garcia-Garcia A., Martin A.M., Arranz L., Martin-Perez D., Torroja C., Sanchez-Cabo F., Mendez-Ferrer S. (2014). The neural crest is a source of mesenchymal stem cells with specialized hematopoietic stem cell niche function. eLife.

[19] Parfejevs V., Antunes A.T., Sommer L. (2018). Injury and stress responses of adult neural crest-derived cells. Dev. Biol..

[20] Pinho S., Lacombe J., Hanoun M., Mizoguchi T., Bruns I., Kunisaki Y., Frenette P.S. (2013). PDGFRalpha and CD51 mark human nestin+ sphere-forming mesenchymal stem cells capable of hematopoietic progenitor cell expansion. J. Exp. Med..

[21] Xiao Y., McGuinness C.S., Doherty-Boyd W.S., Salmeron-Sanchez M., Donnelly H., Dalby M.J. (2022). Current insights into the bone marrow niche: From biology in vivo to bioengineering ex vivo. Biomaterials.

[22] Wong A., Ghassemi E., Yellowley C.E. (2014). Nestin expression in mesenchymal stromal cells: Regulation by hypoxia and osteogenesis. BMC Vet. Res..

[23] Kretsovali A., Hadjimichael C., Charmpilas N. (2012). Histone deacetylase inhibitors in cell pluripotency, differentiation, and reprogramming. Stem Cells Int..

[24] Lee M.J., Kim Y.S., Kummar S., Giaccone G., Trepel J.B. (2008). Histone deacetylase inhibitors in cancer therapy. Curr. Opin. Oncol..

[25] Chung Y.S., Kim H.J., Kim T.M., Hong S.H., Kwon K.R., An S., Park J.H., Lee S., Oh I.H. (2009). Undifferentiated hematopoietic cells are characterized by a genome-wide undermethylation dip around the transcription start site and a hierarchical epigenetic plasticity. Blood.

[26] Oh I.H., Humphries R.K. (2012). Concise review: Multidimensional regulation of the hematopoietic stem cell state. Stem Cells.

[27] Manocha E., Consonni A., Baggi F., Ciusani E., Cocce V., Paino F., Tremolada C., Caruso A., Alessandri G. (2022). CD146^+^ Pericytes Subset Isolated from Human Micro-Fragmented Fat Tissue Display a Strong Interaction with Endothelial Cells: A Potential Cell Target for Therapeutic Angiogenesis. Int. J. Mol. Sci..

[28] Itkin T., Gur-Cohen S., Spencer J.A., Schajnovitz A., Ramasamy S.K., Kusumbe A.P., Ledergor G., Jung Y., Milo I., Poulos M.G. (2016). Distinct bone marrow blood vessels differentially regulate haematopoiesis. Nature.

[29] Sa da Bandeira D., Casamitjana J., Crisan M. (2017). Pericytes, integral components of adult hematopoietic stem cell niches. Pharmacol. Ther..

[30] Armulik A., Genove G., Betsholtz C. (2011). Pericytes: Developmental, physiological, and pathological perspectives, problems, and promises. Dev. Cell.

[31] Lee H.R., Lee G.Y., Kim E.W., Kim H.J., Lee M., Humphries R.K., Oh I.H. (2022). Reversible switching of leukemic cells to a drug-resistant, stem-like subset via IL-4-mediated cross-talk with mesenchymal stroma. Haematologica.

[32] Jeon S., Lee H.S., Lee G.Y., Park G., Kim T.M., Shin J., Lee C., Oh I.H. (2017). Shift of EMT gradient in 3D spheroid MSCs for activation of mesenchymal niche function. Sci. Rep..

[33] Stevanovic M., Drakulic D., Lazic A., Ninkovic D.S., Schwirtlich M., Mojsin M. (2021). SOX Transcription Factors as Important Regulators of Neuronal and Glial Differentiation During Nervous System Development and Adult Neurogenesis. Front. Mol. Neurosci..

[34] Mehrotra P., Tseropoulos G., Bronner M.E., Andreadis S.T. (2020). Adult tissue-derived neural crest-like stem cells: Sources, regulatory networks, and translational potential. Stem Cells Transl. Med..

[35] Duvic M., Vu J. (2007). Vorinostat in cutaneous T-cell lymphoma. Drugs Today.

[36] Duvic M., Vu J. (2007). Update on the treatment of cutaneous T-cell lymphoma (CTCL): Focus on vorinostat. Biologics.

[37] Prebet T., Vey N. (2011). Vorinostat in acute myeloid leukemia and myelodysplastic syndromes. Expert Opin. Investig. Drugs.

[38] Jeong S.Y., Kim J.A., Oh I.H. (2018). The Adaptive Remodeling of Stem Cell Niche in Stimulated Bone Marrow Counteracts the Leukemic Niche. Stem Cells.

[39] Kim J.A., Shim J.S., Lee G.Y., Yim H.W., Kim T.M., Kim M., Leem S.H., Lee J.W., Min C.K., Oh I.H. (2015). Microenvironmental remodeling as a parameter and prognostic factor of heterogeneous leukemogenesis in acute myelogenous leukemia. Cancer Res..

[40] Hu C., Li L. (2018). Preconditioning influences mesenchymal stem cell properties in vitro and in vivo. J. Cell Mol. Med..

[41] Pawitan J.A., Bui T.A., Mubarok W., Antarianto R.D., Nurhayati R.W., Dilogo I.H., Oceandy D. (2020). Enhancement of the Therapeutic Capacity of Mesenchymal Stem Cells by Genetic Modification: A Systematic Review. Front. Cell Dev. Biol..

[42] Sarkar D., Vemula P.K., Teo G.S., Spelke D., Karnik R., Wee L.Y., Karp J.M. (2008). Chemical engineering of mesenchymal stem cells to induce a cell rolling response. Bioconjug. Chem..

[43] Yin J.Q., Zhu J., Ankrum J.A. (2019). Manufacturing of primed mesenchymal stromal cells for therapy. Nat. Biomed. Eng..

[44] Ekins S., Williams A.J., Krasowski M.D., Freundlich J.S. (2011). In silico repositioning of approved drugs for rare and neglected diseases. Drug Discov. Today.

[45] He B., Hou F., Ren C., Bing P., Xiao X. (2021). A Review of Current In Silico Methods for Repositioning Drugs and Chemical Compounds. Front. Oncol..

[46] Burja B., Barlič A., Erman A., Mrak-Poljšak K., Tomšič M., Sodin-Semrl S., Lakota K. (2020). Human mesenchymal stromal cells from different tissues exhibit unique responses to different inflammatory stimuli. Curr. Res. Transl. Med..

[47] Hass R., Kasper C., Böhm S., Jacobs R. (2011). Different populations and sources of human mesenchymal stem cells (MSC): A comparison of adult and neonatal tissue-derived MSC. Cell Commun. Signal..

[48] Kern S., Eichler H., Stoeve J., Klüter H., Bieback K. (2006). Comparative analysis of mesenchymal stem cells from bone marrow, umbilical cord blood, or adipose tissue. Stem Cells.

[49] Iancu-Rubin C., Hoffman R. (2015). Role of epigenetic reprogramming in hematopoietic stem cell function. Curr. Opin. Hematol..

