# Supplementary material for: Chemical Coaxing of Mesenchymal Stromal Cells by Drug Repositioning for Nestin Induction
Source: Int J Mol Sci. 2024 Jul 23;25(15):8006. doi: 10.3390/ijms25158006 (PMC11311338; doi:10.3390/ijms25158006)
Supplement: Supplementary file 1 [file ijms-25-08006-s001.zip › ijms-3075573-supplementary.pdf]

Supplementary Data

Chemical Coaxing of Mesenchymal Stromal Cells by Drug Repositioning for Nestin Induction

Sun-Ung Lim [1], Dae-Won Lee [1], Jung-Ho Kim [2], Young-Ju Kang [2], In-Yong Kim [1] and Il-Hoan Oh [1,2,*]

1 Catholic High-Performance Cell Therapy Center & Department of Medical Life Science, College of Medicine, The Catholic University of Korea, 222, Banpo-Daero, Seocho-Gu, Seoul 06591, Republic of Korea; woong@catholic.ac.kr (S.-U.L.); lovebauoo@catholic.ac.kr (D.-W.L.); iykim@catholic.ac.kr (I.-Y.K.)
2 Regen Innopharm Inc., Seoul 06591, Republic of Korea; leeluleel@catholic.ac.kr (J.-H.K.); yjkang@regeninnopharm.com (Y.-J.K.)
* Correspondence: iho@catholic.ac.kr; Tel.: +82-2-3147-8973

Table S1. Chemicals inducing nestin expression, approved by U.S. FDA

No.	Structure	Source	Dose (*in vitro*)	% FACS[1]
1	Vorinostat 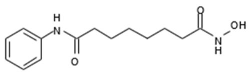	MicroSource	10 µM	89.0
2	Reserpine 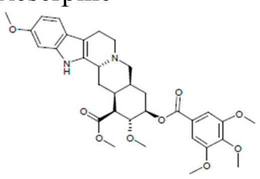	MicroSource	10 µM	91.6
3	CY 208-243 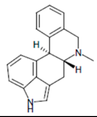	Tocris	10 µM	98.2
4	Elacridar 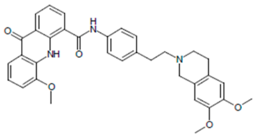	Chemscene	10 µM	97.9
5	Triamcinolone 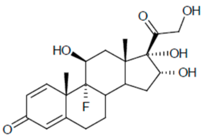	MicroSource	10 µM	86.0

Chemicals were obtained from Korea Chemical Bank of Korea Research Institute of Chemical Technology.

[1] % FACS for GFP positive MSCs.

Table S2. Primer information used in Real-time PCR

Gene	Primer	Sequence
NESTIN	Forward	AGT GAT GCC CCT TCA CCT TG
	Reverse	GCT CGC TCT CTA CTT TCC CC
CD146	Forward	TCC ACA GAG AGA AAG CTG CC
	Reverse	GCT CTT ACG AGA CGG GGG TA
PDGFR-B	Forward	TAG TGT TCG AGG CCT ACC CA
	Reverse	CAG CTC TGA CAC ATA CCG GG
NG2	Forward	CAC TCA GGC AGA GGT CTA CG
	Reverse	GCA GCC TCA AAA GAC ACA GC
SNAI1	Forward	GCG AGC TGC AGG ACT CTA AT
	Reverse	GGA CAG AGT CCC AGA TGA GC
TWIST1	Forward	TGC CAA TCA GCC ACT GAA AGG
	Reverse	TTT GCA GGC CAG TTT GAT CCC
ZEB1	Forward	TGC ACT GAG TGT GGA AAA GC
	Reverse	TGG TGA TGC TGA AAG AGA CG
NANOG	Forward	CAA AGG CAA ACA ACC CAC TT
	Reverse	TCT GCT GGA GGC TGA GGT AT
SOX2	Forward	GCT ACA GCA TGA TGC AGG ACC A
	Reverse	TCT GCG AGC TGG TCA TGG AGT T
OCT4	Forward	TTC AGC CAA ACG ACC ATC TG
	Reverse	CAC GAG GGT TTC TGC TTT GC
SOX9	Forward	TAC GAC TAC ACC GAC CAC CA
	Reverse	CTC CTC AAG GTC GAG TGA GC
SOX10	Forward	GCT GCT GAA CGA AAG TGA CA
	Reverse	AAG TGG GCG CTC TTG TAG TG
MAP2	Forward	CAT CCG CCA CAG GCC AGG TG

	Reverse	GTC GTC GGG GTG ATG CCA CG
TUJ1	Forward	GGC CTC TTC TCA CAA GTA CG
	Reverse	CCA CTC TGA CCA AAG ATG AAA
GAPDH	Forward	CTG GTA AAG TGG ATA TTG TTG CCA T
	Reverse	TGG AAT CAT ATT GGA ACA TGT AAA CC

Table S3. Antibody information used in FACS

Antibody	Company	Fluorescence	Cat. No.
CD146	BD Biosciences	PE-Cy7	562135
CD105	BD Biosciences	APC	562408
CD90	BD Biosciences	FITC	555595
VCAM-1	BD Biosciences	BV605	744309
CD44	BD Biosciences	APC	559942
CD45	BD Biosciences	PE	555483
CD34	BD Biosciences	APC	555824
CD90	BD Biosciences	PE-Cy7	561558

Antibody information used in immunofluorescence and immunohistochemistry

Antibody	Company	Host	Cat. No.
N-F	Biolegend	Mouse	837802
TUJ1	Abcam	Rabbit	ab15461

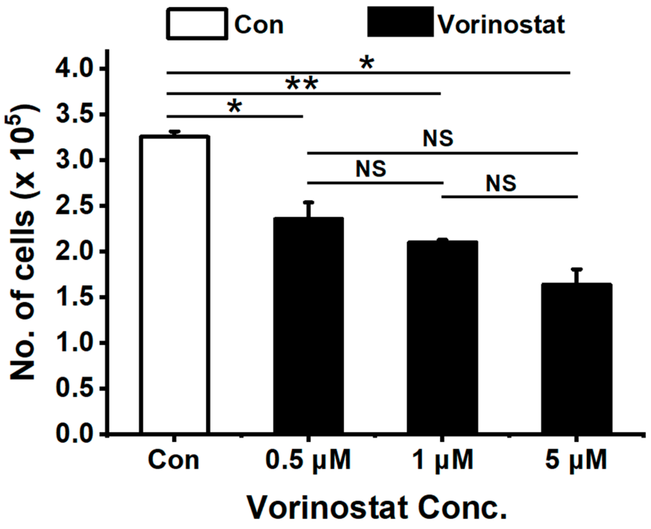

Figure S1. Relative cell number of MSCs after 3 days of exposure to 0.5, 1, and 5 μM of vorinostat. MSCs were cultured in DMEM supplemented with 10% FBS. Data are represented as mean ± SD. *$*p < 0.05$, $**p < 0.01$*.
